# Structural implications of Dpy30 oligomerization for MLL/SET1 COMPASS H3K4 trimethylation

**DOI:** 10.1007/s13238-014-0127-z

**Published:** 2014-12-27

**Authors:** Hongmei Zhang, Mei Li, Yu Gao, Chenjun Jia, Xiaowei Pan, Peng Cao, Xuelin Zhao, Jiping Zhang, Wenrui Chang

**Affiliations:** 1 Institute of Biophysics, Chinese Academy of Sciences, Beijing, 100101 China; 2 University of Chinese Academy of Sciences, Beijing, 100049 China

**Dear Editor,**

The methylation modifications of histone 3 lysine 4 (H3K4) have essential effects on biological processes including gene expression and transcription, cell cycle progression, and DNA repair. From yeast to mammals, the SET1 and MLL-like (mixed-lineage leukemia) multi-subunit protein complexes, known as SET1 or MLL COMPASS (Schneider et al., 2005), are responsible for H3K4 methylation. In addition to the catalytic SET domain, the SET1 and MLL COMPASS complexes also contain a number of conserved subunits including WDR5 (Cps30), RbBP5 (Cps50), Ash2L (Cps60 or Bre2), and Dpy30 (Cps25 or Sdc1). Dpy30 is an important subunit of MLL/SET1 complexes, where it plays an essential role in both catalyzing H3K4 trimethylation and maintaining the state of trimethylated H3K4. Many regulatory gene loci in undifferentiated embryonic stem cells (ESCs) exhibit both H3K4 and H3K27 methylation modifications, the so-called bivalent marks, which poise the genes for expression (Azuara et al., 2006; Bernstein et al., 2006; Pan et al., 2007). Dpy30 is the subunit that regulates chromosomal H3K4 trimethylation (H3K4me3) throughout the mammalian genome by MLL complexes; deletion of Dpy30 alters the differentiation process of ESCs (Jiang et al., 2011).

Dpy30 directly interacts with Ash2L in human (Bre2 in yeast) COMPASS through its Dpy30-binding motif (DBM) (South et al., 2010). Recent *in vitro* and *in vivo* studies indicate that both Dpy30 and Ash2L are important for the H3K4 trimethylation activity of SET1/MLL complexes (Dou et al., 2006; Patel et al., 2011). Although the structures of the human Dpy30 C-terminal domain (Dpy30C) and SPRY domain of Ash2L containing the DBM motif were solved (Wang et al., 2009b), the binding mode and stoichiometry of Dpy30 and Ash2L within COMPASS remain uncertain, partly because the complex structure is lacking.

The DBM motif sequences of human Ash2L and yeast Bre2 share 25.9% identity and 46.2% similarity. Also, human Dpy30 and yeast Sdc1 have a conserved Dpy30 domain of about 41 amino acids with 37% identity and 74% similarity. To investigate the interaction between Dpy30 and Ash2L/Bre2, we synthesized their DBM motifs (Ash2L_DBM_ and Bre2_DBM_), measured their binding affinity with Dpy30FL as well as Dpy30C using isothermal titration calorimetry (ITC). We found that the *K*d values are very similar (Fig. 1A–C), indicating that the two peptides have a similar interacting affinity with Dpy30. The ITC results also confirm the previous report (South et al., 2010) that Dpy30C is the region of the Dpy30 protein that binds the DBM motif.

**Figure 1:**
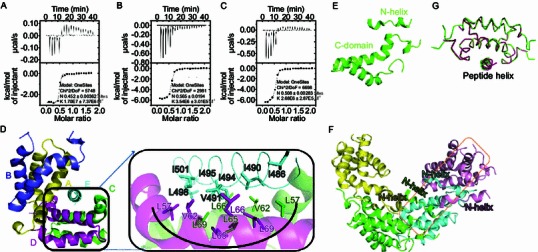
ITC analyses and structural representations of Dpy30C complexed with Bre2_DBM_ and Dpy30FL. (A–C) Isothermal titration calorimetry evaluation of the interaction between Dpy30FL and Ash2L_DBM_ peptide (panel A), Dpy30C and Bre2_DBM_ peptide (panel B), and Dpy30C and the Ash2L peptide (panel C). (D) Overall structure of the Dpy30C in complex with Bre2_DBM_ (Dpy30C-Bre2_DBM_). The left panel shows the location of Bre2_DBM_. The right panel shows a cartoon-stick model of the symmetric and hydrophobic interactions between the Bre2_DBM_ peptide and Dpy30C. Chains A, B, C, D, and E are yellow, purple, green, magenta, and cyan, respectively. (E) Monomer structure of Dpy30FL. The C-domain and N-helix are labeled. The part between the C-terminal domain and N-terminal helix, which is not traced in the present structure, is shown as a dotted line. (F) Tetramer model of Dpy30FL. Four neighboring Dpy30FL dimers are represented in light purple, cyan, green, and yellow. The N-terminal helix is accommodated by the neighboring Dpy30C domain. Two adjacent dimers could form a tetramer. The region inside the wheat box is a model for one Dpy30C tetramer binding two peptides. (G) Superimposed complex of the Dpy30C dimer with Bre2 peptide onto the Dpy30FL dimer binding the N-terminal helix. The Dpy30FL dimer with N-terminal helix is green, and the Dpy30C dimer binding the Bre2 peptide is red

The attempts to crystallize the Dpy30 complexed with Ash2L_DBM_ peptide were failed. However, the complex of Dpy30C and Bre2_DBM_ was crystallized successfully and the structure (Dpy30C-Bre2_DBM_) was solved to 2.15 Å resolution (Table S1). Considering the sequence similarity of the two peptides, we believe that the structural model of Dpy30 complexed with the Bre2_DBM_ should represent the interactional pattern between Dpy30 and Ash2L_DBM_. The crystal structure of Dpy30C-Bre2_DBM_ contains two dimers of Dpy30C (chain A and B: AB dimer; chain C and D: CD dimer) and only one copy of Bre2_DBM_ (chain E) in an asymmetrical unit (Fig. 1D). The overall structure of the complex fits the electronic density well except the DBM peptide. It is difficult to refine the Bre2_DBM_ because there is always some discontinuous residual density around the DBM peptide in the electronic density difference map for the final model. After multiple refining trials, we determined that the residual density could be fitted by a similar helix with a reversed orientation. Finally, two separated structures, with the orientation of the helix different from each other, were refined and showed similar qualities with *R*_work_/*R*_free_ of 0.18/0.21 and 0.18/0.22 (Fig. S1A and S1B), respectively. It is likely that the structure represents a mixed model, which was led by the fact that some DBM helices were bound in one orientation and the rest were in a reversed orientation in the complex crystal (Fig. S1C). In the two types of structures, the DBM peptide interacts with Dpy30C in the same mode and position, although in opposite orientation. According to these results, we used the complex model with the higher quality for the structural comparison and discussion.

The Bre2_DBM_ peptide interacts simultaneously with two Dpy30C dimers, albeit with different affinities. The CD dimer plays a dominant role in Bre2_DBM_ binding with a buried area of about 836.2 Å^2^, while the AB dimer assists in the interaction, forming a binding area of about 292.5 Å^2^ with Bre2. The interactions between Dpy30C and Bre2_DBM_ are mainly hydrophobic without specific hydrogen-bond or salt-bond interactions. Ten hydrophobic residues from chains C and D of the Dpy30C dimer are distributed symmetrically on the inner surface in a semi-circular shape, forming a hydrophobic groove (Fig. 1D). Simultaneously, the Bre2_DBM_ helix also shows an amphiphilic characteristic. Centering on Trp492, its hydrophobic residues arranged symmetrically, facing toward the two Dpy30C monomers. This binding pattern, together with the symmetric properties of both the Bre2_DBM_ peptide sequence and the residues in the hydrophobic groove of Dpy30C, might be the reason that the Bre2_DBM_ peptide can bind to the Dpy30C dimer in reversed orientations without any preference. We also solved the Dpy30 full-length (Dpy30FL) structure at a resolution of 2.13 Å (Table S1). It contains two Dyp30 molecules in an asymmetric unit. Although we used the Dpy30FL sample for crystallization, only the C-terminal 55 residues (amino acids 45–99) were well defined in the structure. The C-terminal domains of two Dpy30 monomers form a semi-circle hydrophobic groove, accommodating a helix with poorer electron density. We can only model a fragment of poly-alanine here without visible side chains based on the electron density map. We deduced that this fragment could be the Dpy30 N-terminal domain because no other peptide was mixed with the protein during purification and crystallization. It seems that the N-terminal domain of Dpy30 forms an N-helix that connects to the C-terminal domain through a flexible loop that is also missing in the structure (Fig. 1E). In the Dpy30FL dimer, two C-terminal domains fold as a four-helix bundle and are packed tightly, while the N-helix swings randomly in solution.

The structural characteristics of the C-terminal domain in the Dpy30FL structure are the same as those in the Dpy30-Bre2_DBM_ complex and the previously reported structure (Wang et al., 2009b). One N-terminal helix was bound in the hydrophobic groove of the neighboring Dpy30C dimer (Fig. 1F). Curiously, both the SPR and ITC experimental results indicated that the Dpy30C domain had no interaction with the Dpy30 N-helix in solution. It is possible that the N-helix residues artificially in the hydrophobic groove of the Dpy30C dimer because of molecular packing in the crystal. However, it is noteworthy that the binding site is exactly the same as that observed for the Dpy30C dimer (CD) interacting with the Bre2_DBM_ peptide. Superimposition of the Dpy30FL model and the model of the Dpy30C dimer (CD) binding the Bre2_DBM_ peptide (Fig. 1G) showed that, not only the Dpy30-C dimers themselves matched very well, but also the bound N-helix and the Bre2 peptide, with a root-mean-square deviation of 0.6 Å. This result suggests that the bound N-helix occupies the binding site of the target protein of Dpy30FL in crystal. Based on the two structures above, we presented a structural model of Dpy30C in complex with its target helix in a 2:1 ratio, as shown in Fig. 1G. This could be the basic unit for Dpy30 binding to Ash2L or Bre2.

The molecular packing pattern of the Dpy30FL crystal indicates that Dpy30 oligomerizes with weak stability. The Dpy30FL crystals belong to space group P4_1_2_1_2. Molecules are packed along the 4_1_ screw axis to form a helical polymer that has a width of ~45 Å (Fig. S2A). The basic structure of the helical polymer is a Dpy30 dimer, each turn of the helical polymer includes four dimers, and any two neighboring dimers are 1/4 units apart along the spiral axis and form a separation angle of 90° (Fig. S2B). Two neighboring dimers form a tetramer (Fig. 1F) that is stabilized by weak hydrophobic interactions. The buried area of two adjacent dimers is 909.8 Å^2^, and the complexation significance score (CSS) value is 0.841 calculated using PISA software (http://www.ebi.ac.uk/msd-srv/prot_int/pistart.html). Considering the intermolecular interaction area of 1159.3 Å^2^ and a CSS value of 0.867 within the dimer, the intra- and inter-dimer interaction forces are approximately identical. In the Dpy30FL structure, a Dpy30C dimer binds with one target peptide; thus, the packing pattern presents a hexamer model of Dpy30 interacting with target helices at a ratio of 4:2.

In the Dpy30-Bre2_DBM_ complex structure, two Dpy30 dimers form a loose tetramer with a crossing angle of 60° (Fig. 1D). The interface is formed between residues 47 and 71 of the neighboring chains A and D, with the buried area occupying 549 Å^2^. Bre2_DBM_ binding to Dpy30C increases this area to 1128.7 Å^2^, which stabilizes the Dpy30C tetramer. This structure represents a model of Dpy30 binding to the target helix at the ratio of 4:1. Combined with the model of one Dpy30 tetramer binding two peptides, these results suggest that Dpy30 can interact with its protein partners, such as Ash2L, at different stoichiometric values. The state of Dpy30 binding target peptide is referred to as holo-Dpy30.

A previously reported crystal structure of Dpy30C (PDB code 3G36) was analyzed here as a tetramer. The Dpy30C structure contained two dimers (A and C, and B and D) with an anti-parallel arrangement in an asymmetric unit (Fig. 2A). The two dimers form strong interactions with an interface area of 947.3 Å^2^, and the CSS value of the neighboring dimers is 0.723. In addition, the quaternary structure assembly of Dpy30C represents a stable tetramer or octamer, as analyzed using PISA software. In the tetramer, the contact surface was composed of residues 57–76 from chains A and B in both dimers. Two hydrogen-bond clusters between two dimers are important to stabilize the tetramer. The residues T53 and R54 form as many as nine hydrogen bonds with residues R54, D58, and Q59 from the adjacent dimer (Fig. 2A). The Dpy30C tetramer packed too close to accommodate one target helix (referred to as apo-Dpy30C).

**Figure 2:**
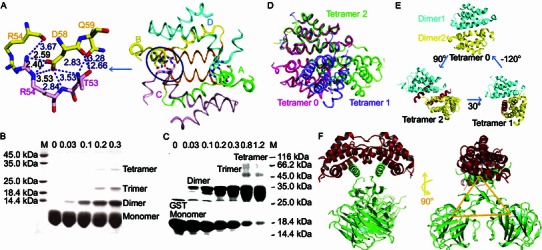
Dpy30 is able to form tetramer and interact with other protein partners in its tetrameric state. (A) Cartoon and stick model of the apo-Dpy30C tetramer (PDB code 3G36). In the right panel, the orange region (shown as cartoon helix) represents the main interface between two neighboring dimers. The hydrogen-bond clusters (shown as stick models) flank the two sides of this region. In the left panel, a stick model shows the details of the hydrogen-bond clusters made by R54 and T53 of chain C (light pink) connecting with R54, D58, and Q59 of chain B (yellow) (the same hydrogen-bond clusters are also formed between chains D and A). The hydrogen bond interactions are shown as dashed lines with distances indicated. (B and C) A cross-linking assay of Dpy30C and Dpy30FL was analyzed by 15% SDS-PAGE and are presented in panel (B) and (C), respectively. Lanes from left to right contain bis(sulfosuccinimidyl) suberate with increasing concentrations from 0 to 1.2 mmol/L. (D) Superimposition of the three different Dpy30C tetrameric forms. Tetramers 0, 1, and 2, representing models for Dpy30 in complex with zero, one, and two target proteins, are shown in light pink, light purple, and green, respectively. (E) Movement between the two dimers from an apo-state to holo-state of Dpy30 tetramer. Dimer 1 is cyan and dimer 2 is yellow for all three forms of the Dpy30C tetramer. The Bre2_DBM_ peptide is firebrick. (F) Model of tetrameric Dpy30C (shown in firebrick) bound to the dimeric SPRY domain (PDB code: 3TOJ, shown in green). The model on the left shows a triangular conformation with three similar-sized lobes representing one tetrameric Dpy30 and one dimeric SPRY domain. At the center of the triangular region are four interacting β-strands from the dimeric SPRY domain. On the right, the model is turned 90° around its y-axis to better illustrate the interaction between the SPRY helices and the Dpy30 tetramer

Although analysis of static light scattering and analytical ultra-centrifugation showed that in solution, both Dpy30FL and Dpy30C are present in their dimeric forms (Fig. S3), the crystal structures of both holo-Dpy30 and apo-Dpy30C, as well as the previous report (van Nuland R., 2013), all suggest the possibility of Dpy30 oligomerization. Thus, we performed biochemical analysis to investigate the oligomeric state of Dpy30 in solution. Cross-linkage assays using bis(sulfosuccinimidyl) suberate (BS3) reagent indicated that the majority of both the Dpy30 C-terminal domain and the Dpy30FL molecules form dimers, while a small percentage of molecules forms trimers, tetramers, and higher-order oligomers. The monomeric bands become weaker, while the dimeric, trimeric, and tetrameric bands become stronger as a function of increasing concentrations of BS3 (Fig. 2B and 2C).

Three models of the Dpy30 tetramer with or without its target peptide are now available. Superimposing the three Dpy30 tetramer models showed that the dimeric structures are almost identical (Fig. 2D). However, the relative position between the two dimers differs substantially among the three models. In the two structures of Dpy30 bound with target peptides, dimer 2 makes a movement away from dimer 1, simultaneously rotating 90° and 60°, breaking the hydrogen-bond interactions in the apo-Dpy30 state. As an important consequence of this movement, the space between dimers opens wide enough to accommodate two and one target peptide, respectively, and the conformation of the Dpy30 tetramer switches from apo-Dpy30 to holo-Dpy30 (Fig. 2E).

Our ITC results showed that the stoichiometric value N for Dpy30 binding Bre2_DBM_ and Ash2L_DBM_ were all close to 0.5 (Fig. 1A–C), indicating that the ratio of Dpy30 to Ash2L or Bre2 in solution is 2:1, which is consistent with the previous results (Wang et al., 2009a; Chen et al., 2012) and the Dpy30FL structure. Considering Dpy30 can form weak tetramer, it might present as a tetramer and bind two Ash2L in MLL/SET1 COMPASS. It was supported by the previous report that the yeast COMPASS contains two copies of Cps60/Ash2L, and at least three copies of Sdc1/Dpy30 (Schneider et al., 2005). The three-dimensional electron microscopic model of human and yeast SET/MLL COMPASS displayed two subunits, Cps60/Ash2L and Cps25/Dpy30, as three lobes of similar size forming a triangular base structure (Takahashi et al., 2011). Since the apparent molecular weight of the Dpy30 tetramer is about equal to the molecular weight of an Ash2L monomer, we hypothesize that one Dpy30 tetramer could bind one Ash2L dimer within COMPASS. The SPRY domain containing a DBM motif of Ash2L was structurally characterized recently (Chen et al., 2012). The analysis of the SPRY structure indicates that there are 10 pairs of hydrogen bonds and one disulfide bond at the interface between two monomers of the SPRY domain, and analysis using PISA software suggests that the SPRY domain can form stable dimers in solution, supporting our hypothesis that the Dpy30 tetramer interacts with an Ash2L dimer within COMPASS.

Combining the structural and biochemical data, we constructed a Dpy30-Ash2L complex model using the structures of the holo-Dpy30FL tetramer presented here and the SPRY dimer reported previously (3TOJ). In this model, one copy of the Dpy30 tetramer and two copies of the Ash2L monomer occupy the three lobes of the triangular base of MLL/SET1 COMPASS (Fig. 2F). Two Dpy30 dimers form a weak tetramer in this model; each dimer binds one Ash2L, with two Ash2L molecules forming a weak dimer. Because of the formation of multi-contacting surfaces in this complex model, we believe that the hetero-hexameric form is more stable than the Ash2L dimer or Dpy30 tetramer alone, and that it could represent one possible state of Dpy30 interacting with Ash2L in COMPASS.

Recently, Timmers et al. observed that Dpy30 interacted with Ash2L with various ratios in different COMPASS complexes. The ratio is 6:1 in the MLL1/2 complex (van Nuland R., 2013), which is necessary for H3K4me3 (Wu et al., 2008; Wang et al., 2009a); 3:1 in the MLL3/4 complex (van Nuland R., 2013), which is critical for mono-methylation (Herz et al., 2012); and 2:1 or 3:1 in the SETA/B complex, which is responsible for the global levels of H3K4me3 (Lee et al., 2007; Wu et al., 2008). This suggests that Dpy30 exists as various oligomers of a genuine subunit in different MLL/SET complex members. It seems that H3K4me3 requires more copies of Dpy30 than H3K4me and H3K4me2. Based on our structural data and cross-linking results, we suggest that Dpy30 may regulate H3K4 methylation according to its copy number in COMPASS. However, more cytological and biochemical experiments are required to fully understand the precise function of Dpy30 oligomerization.

## Electronic supplementary material

Below is the link to the electronic supplementary material.

Supplementary material 1 (PDF 467 kb)
