# Supplementary material for: Structural implications of Dpy30 oligomerization for MLL/SET1 COMPASS H3K4 trimethylation
Source: Protein Cell. 2014 Dec 27;6(2):147–51. doi: 10.1007/s13238-014-0127-z (PMC4312765; doi:10.1007/s13238-014-0127-z)
Supplement: Supplementary file 1 — Supplementary material 1 (PDF 467 kb) [file 13238_2014_127_MOESM1_ESM.pdf]

Table 1 Data collection and refinement statistics

**Data collection** [a]	Dpy30FL	Dpy30C-Bre$_{DBM}$
Space group	$P4_12_12$	$P6_1$
Cell dimensions (Å)	89.6,89.6,53.2,	75.2,75.2,105.8,
	90,90,90	90,90,120
Rmerge (%)[b]	10.3 (63.3)	6.9 (39.6)
I/sigma	24.8 (5)	35 (6.9)
Resolution (Å)	30-2.13	50-2.15
Completeness (%)	99.6 (100)	100 (100)
Redundancy	10 (11.1)	11.3 (11.4)
Wavelength (Å)	0.97872	0.97872
**Refinement**		
No. Reflections	12624	22964
Rwork/Rfree (%)[c]	20.3/22.7	17.8/21.3
Resolution (Å)	27.2-2.13	35-2.15
B-factor ($Å^2$)		
all	49.6	45.4
protein	49	46.89
peptide		73.74
waters	48	54.97
FMT	35.0	
RMSD bond lengths (Å)	0.007	0.011
RMSD bond angles (°)	1.005	1.325
**Ramachandran plot**		
Most favored regions (%)	97.6	98.2
Allowed regions (%)	2.4	1.8

Disallowed regions (%)	0.0	0.0
**PDB accession code**	4RTA	4RT4

[a] Figures in brackets indicate the values for the outer resolution shell.

[b] $R_{merge} = \Sigma_h \Sigma_l |I_{hl} - \langle I_h \rangle| / \Sigma_h \Sigma_l \langle I_h \rangle$, where I_l is the lth observation of reflection h, and $\langle I_h \rangle$ is the weighted average intensity for all observations l of reflection h.

[c] R-factors and Rfree were calculated as follows: $R = \Sigma (|F_{obs} - F_{calc}|)/\Sigma |F_{obs}| \times 100$, where Fobs and Fcalc are the observed and calculated structure factor amplitudes, respectively.

Figure S1 Electron density map for the peptide of Dpy30-binding motif (DBM) in Bre2. *A.* 2*Fo-Fc* electron density map (contour at 1σ) of the helix with the forward orientation. *B.* 2*Fo-Fc* electron density map (contour at 1σ) of the helix with the reversed orientation. Each of the helices can be fitted into the complex structure of Dpy30 with $\mathrm{Bre2}_{\mathrm{DBM}}$ and further refined. *C.* 2*Fo-Fc* electron density map (blue, contour at 2σ) and *Fo-Fc* electron density map (green, contour at 3.5σ) show that the DBM peptide, with two different orientations (blue and dark green), co-occupy the same location. For clarity, only the Cα chains of the peptides were shown.

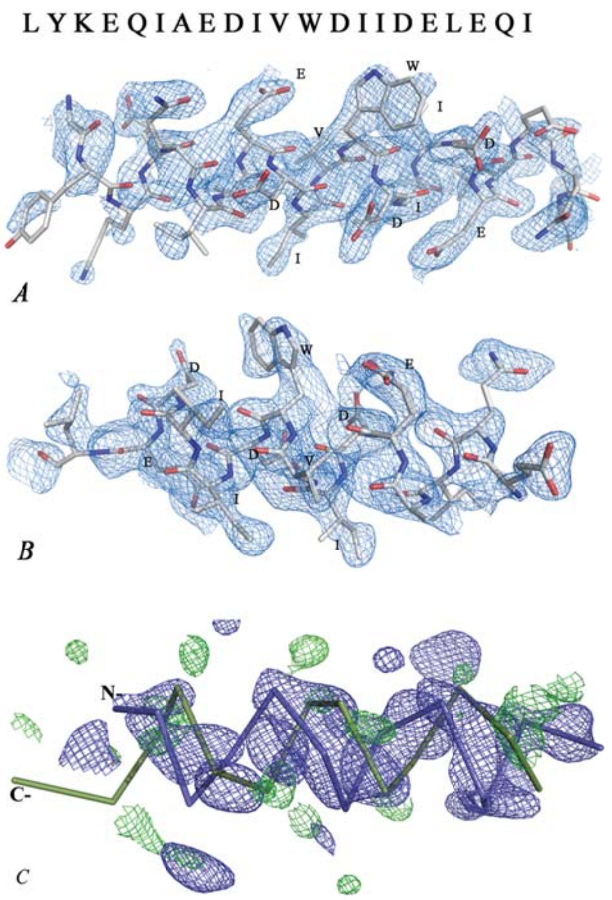

Figure S2 *A*. Molecular packing of Dpy30FL molecules in crystal. The packing pattern shows helical polymers formed by Dpy30FL molecules in crystal. The two monomers of the reference dimers are green and magenta, and other molecules are symmetry mates. *B*. Four dimers spiraling around the 4_1 screw axis are yellow, green, cyan and light purple. The N-terminal helix of one monomer is located on the neighboring dimer.

A *B*

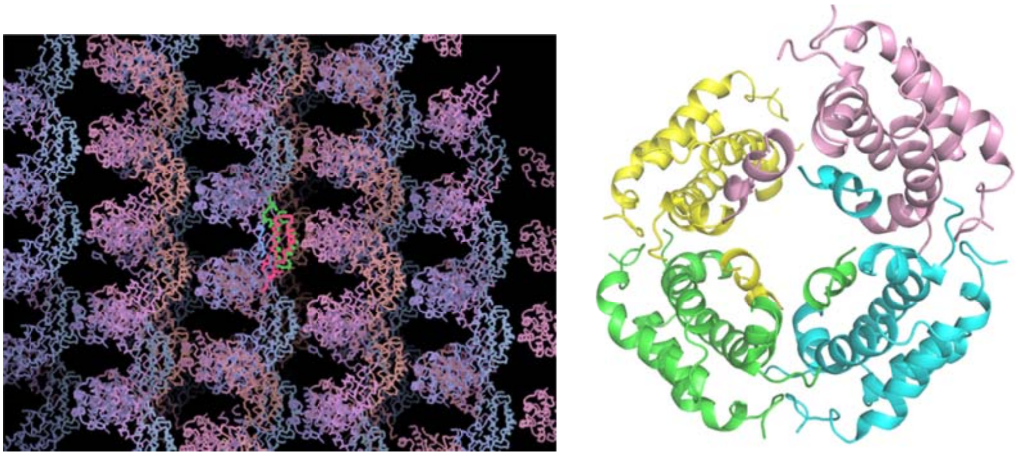

Figure S3 Analysis of molecular mass distributions by analytical ultracentrifugation. Molecular weights of full-length Dpy30 (Dpy30FL), Dpy30 C-terminal domain (Dpy30C) and Dpy30C with the Bre2 peptide are indicated.

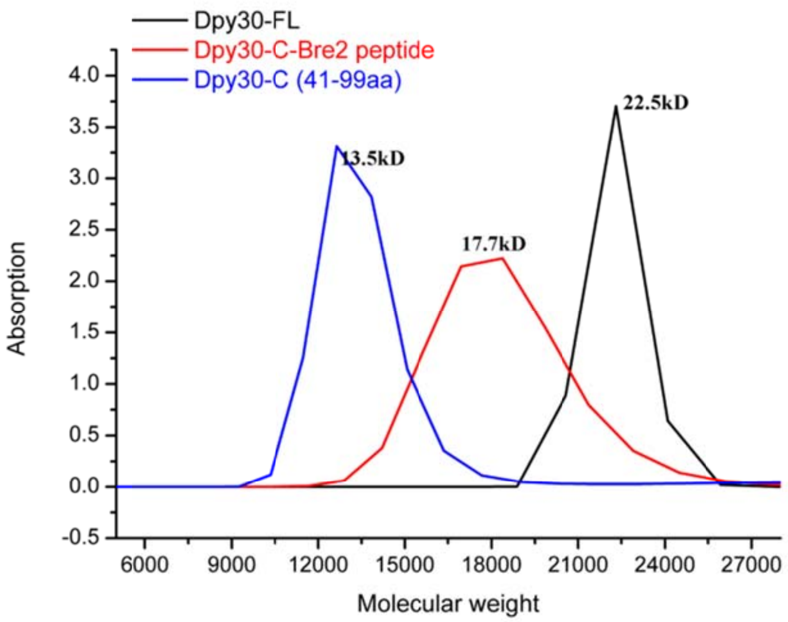

Material and Methods

Cloning, expression, and purification of proteins

Dpy30FL and Dpy30C (amino acids 40–99) were cloned into PGEX-6p-1 vectors (26 kDa). Proteins with glutathione S-transferase (GST) tags were expressed in BL21 (DE3) cells after induction with 0.5 mM isopropyl β-D-1-thiogalactopyranoside for 3 hours at 37°C. Harvested cells were lysed and purified with Glutathione Sepharose Affinity resin (Generon), and the GST tag was removed using precision protease in a buffer containing 50 mM Tris-HCl, pH 8.0, 1 mM DTT, 1 mM EDTA, and 150 mM NaCl. Dpy30FL was further purified by anion exchange on a Resource Q column (GE Healthcare) and subsequent gel filtration on Superdex 75 10/300GL (GE Healthcare). The purified protein was concentrated to 30 mg/mL for crystallization. Dpy30C without a GST-tag was purified by cation exchange on a Resource S column (GE Healthcare) followed by gel filtration on Superdex 75 10/300GL using the same buffer as that for Dpy30FL. Buffers contained 50 mM cacodylate (pH 5.9) for ion exchange and 1× PBS for gel filtration. Purified Dpy30C was mixed with $Bre2_{DBM}$ peptides at a molar ratio of 2:1 and incubated at 4°C overnight. The Dpy30C-$Bre2_{DBM}$ complex eluted from the Superdex 75 column was concentrated to 15 mg/mL for crystallization.

Crystallization, data collection, and structure determination

Initial crystallization trials were carried out at 18°C by mixing 1 μL protein and 1 μL mother liquid using the sitting-drop diffusion method. Dpy30FL crystals appeared initially in condition number 33 of the crystal screen suite (Hampton) and were then

optimized manually. The final crystallization and cryo-protection formulations were pH 5.9, 0.1 M sodium cacodylate, and sodium formate ranging from 3.9 to 5.2 M. Crystals of Dpy30C in complex with $Bre2_{DBM}$ were grown in a solution of 0.1 M Tris-HCl, pH 8.5, 2.0 M $NH_4H_2PO_4$ for more than half a year. The Dpy30FL crystal was directly flash-cooled without additional cryoprotection in a nitrogen-gas stream at 100 K for data collection. The Dpy30C complex crystal was cryoprotected using 80% solution of crystallization and 20% glycerol.

Crystal data at a resolution of 2.1 Å for both Dpy30FL and Dpy30C in complex with $Bre2_{DBM}$ were collected at the Shanghai Light Synchrotron Radiation Facility (Shanghai, PR China) on station BL17U and processed by HKL2000 (Otwinowski, 1997). Data collection statistics are summarized in Supplementary Table 1. Dpy30FL and Dpy30C-$Bre2_{DBM}$ complex crystals belong to space groups $P4_12_12$ and $P6_1$, respectively. The Dpy30FL structure was solved by molecular replacement with Phaser (McCoy, 2007) using the apo-Dpy30C structure (PDB code 3G36) as a search model. The Dpy30C complex structure was solved by molecular replacement using the C-terminal domain of Dpy30FL as a search model. Difference maps were used to rebuild and extend the initial model using the program Coot (Emsley and Cowtan, 2004). Iterative cycles of refinement were carried out using the phenix.refine module of Phenix (Adams et al., 2002). The quality of the overall model was evaluated with Procheck in the ccp4i package (Laskowski RA, 1993). Refinement and evaluation data are summarized in Table 1. Figures in this manuscript were prepared using PyMOL (WL, 2002).

ITC assay

(South et al., 2010) reported that yeast Bre2 and human Ash2L bind Dpy30 through their C-terminal DBM (South et al., 2010). $Bre2_{DBM}$ and $Ash2L_{DBM}$ peptides were ordered from Qiangyao Company (Shanghai) with 95–99% purity as evaluated by mass spectrometry. Final peptide concentrations used for ITC experiments were 650–800 μM, and concentrations of Dpy30FL and Dpy30C were 60–80 μM in 1× PBS. ITC data were analyzed and fitted using Origin 7 (OriginLab).

Dpy30C cross-linking experiments

BS3 was dissolved in 1× PBS at a concentration of 10 mM. The concentration of Dpy30 was 3.7 mg/mL in 1× PBS. Cross-linker was added to six Dpy30 protein samples with final concentrations of 0, 0.03, 0.1, 0.3, 0.8, and 1.2 mM. Reaction mixtures were incubated at room temperature for 30 min before 1× loading buffer was added to quench the reaction. Samples (15 μL) were separated by 15% sodium dodecyl sulfate polyacrylamide gel electrophoresis (SDS-PAGE).

References

Adams, P.D., Grosse-Kunstleve, R.W., Hung, L.W., Ioerger, T.R., McCoy, A.J., Moriarty, N.W., Read, R.J., Sacchettini, J.C., Sauter, N.K., and Terwilliger, T.C. (2002). PHENIX: building new software for automated crystallographic structure determination. Acta Crystallogr D Biol Crystallogr 58, 1948-1954.

Emsley, P., and Cowtan, K. (2004). Coot: model-building tools for molecular graphics.

Acta Crystallogr D Biol Crystallogr 60, 2126-2132.

Laskowski RA, M., M. W., Moss, D. S., and Thornton, J. M. (1993). PROCHECK: a program to check the stereochemical quality of protein structures. . Appl Crystallogr 26, 283-291.

McCoy, A.J. (2007). Solving structures of protein complexes by molecular replacement with Phaser. Acta Crystallogr D Biol Crystallogr 63, 32-41.

Otwinowski, Z.M., W. (1997). Processing of X-ray Diffraction Data Collected in Oscillation Mode. . Methods Enzymol 276, 20.

South, P.F., Fingerman, I.M., Mersman, D.P., Du, H.N., and Briggs, S.D. (2010). A conserved interaction between the SDI domain of Bre2 and the Dpy-30 domain of Sdc1 is required for histone methylation and gene expression. The Journal of biological chemistry 285, 595-607.

WL, D. (2002). The PyMOL Molecular Graphics System. DeLano Scientific, San Carlos, CA, USA.
